# Establishment and Validation of Nomogram Based on Combination of Pretreatment C-Reactive Protein/Albumin Ratio–EBV DNA Grade in Nasopharyngeal Carcinoma Patients Who Received Concurrent Chemoradiotherapy

**DOI:** 10.3389/fonc.2021.583283

**Published:** 2021-07-15

**Authors:** Zhang-Zan Huang, Wen Wen, Xin Hua, Chen-Ge Song, Xi-Wen Bi, Jia-Jia Huang, Wen Xia, Zhong-Yu Yuan

**Affiliations:** State Key Laboratory of Oncology in South China, Collaborative Innovation Center for Cancer Medicine, Guangdong Key Laboratory of Nasopharyngeal Carcinoma Diagnosis and Therapy, Sun Yat-sen University Cancer Center, Guangzhou, China

**Keywords:** survival, nomogram, nasopharyngeal carcinoma, C-E grade, Epstein-Barr virus DNA, CAR

## Abstract

**Background:**

A higher ratio of pretreatment C-reactive protein/albumin ratio (CAR) is associated with poor prognosis in nasopharyngeal carcinoma (NPC), and Epstein–Barr virus (EBV) DNA level is known to not only participate in the occurrence of nasopharyngeal carcinoma but also affect the development and prognosis of the disease. Herein, we proposed that a combination of both these markers could improve the predictive prognostic ability.

**Methods:**

In all, 842 NPC patients who received concurrent chemoradiotherapy (CCRT) were entered in this study. We collected all patients’ blood samples and EBV DNA copy numbers within one week before any treatment. Receiver operating characteristic (ROC) curve was used to determine the optimal cut-off. We employed the Kaplan–Meier method for survival analyses and the univariate and multivariate analyses (Cox proportional hazards regression model) for statistical analysis. A nomogram was constructed based on multivariate analyses results of the validation set. The model was internally validated using 1000 bootstrap samples to avoid overfitting. Another validation of 10-fold cross-validation was also applied. Calibration curves and concordance index (C-index) were calculated to determine predictive and discriminatory capacity.

**Results:**

In the whole cohort, we observed that higher CAR, EBV DNA level, and CAR-EBV DNA (C-E) grade were associated with shorter overall survival (OS) and distant metastasis-free survival (DMFS) (all P<0.05). In univariate and multivariate analyses, C-E grade was an independent prognostic factor (all P<0.05). In the training set, we gained the similar results with the whole set. According to multivariate analyses of the training set, we constructed a nomogram. The results of bootstrap samples and 10-fold cross-validation showed favorable predictive efficacy. And calibration curves of the model provided credibility to its predictive capability.

**Conclusion:**

C-E grade was confirmed as an independent prognostic predictor in patients with NPC who received CCRT. Higher level of pretreatment C-E grade could signify a higher risk of metastasis and shorter OS. The prognostic nomogram based on C-E grade was dependable in nasopharyngeal carcinoma patients.

## Introduction

Nasopharyngeal carcinoma (NPC) is a relatively rare type of head and neck cancer, and is characterized by its unique ethnic and geographic distributions. Southern China has one of the highest incidence of NPC (1), at 20–30 per 100,000 people (2). In addition, the etiology of NPC is distinct from other carcinomas and is related with the Epstein–Barr virus (EBV) (3). During the last several decades, patient prognosis has improved dramatically owing to advances in disease management (4). The widespread application of intensity-modulated radiotherapy and optimization of chemotherapy strategies have contributed to improved survival with reduced toxicities (5, 6). Concurrent chemoradiotherapy (CCRT) had proven to be superior in many clinical trials and is recommended for patients with locally advanced disease (7–11). Although the trends show that the incidence has declined gradually and the mortality reduced substantially, there are still a considerable number of patients with NPC. It is especially crucial to make an accurate assessment to form a suitable treatment plan. Thus far, the gold standard for evaluating the prognosis of NPC is the Union Internationale Contre le Cancer/American Joint Cancer Committee (UICC/AJCC) TNM staging system (12). However, clinicians have noticed that the prognosis of different patients with the same TNM staging is quite distinct. One explanation for this is that the TNM staging system does not account for patients’ personal conditions. Therefore, finding an individual, integrated, and robust indicator is very essential.

Inflammation is known to be associated with cancer (13–15), as it plays an important role in carcinogenesis and tumor progression (16–19). Researchers have shown that inflammation-based indicators are relevant to prognosis of various cancers such as breast cancer (20), gastric cancer (21), rectal and colorectal cancer (22), and lung cancer (23). With respect to NPC, some studies have verified that the neutrophil-to-lymphocyte ratio (NLR), (24), lymphocyte-to-monocyte ratio (LMR) (25), EBV DNA level (26), and C-reactive protein/albumin ratio (CAR) (27) are prognostic biomarkers. C-reactive protein is an accurate protein generated by liver under systemic inflammation and serum albumin is always seen as signal of nutrition status, therefore, CAR, as a high individual marker, could precisely reflect inflammatory nutritional state of objects. Besides, EBV plays an important role in the etiology of NPC. However, to our best knowledge, no study yet has focused on both CAR and EBV DNA levels. Therefore, CAR-EBV DNA (C-E) grade, which combines both the indices, can reflect patient prognosis from two aspects: etiology and personal condition.

At present, no published studies have reported a predictive marker that integrated CAR and EBV DNA level for overall survival (OS) in NPC patients. In this study, we aimed to establish and explore the prognostic ability of CAR-EBV DNA (C-E) grade in patients who received CCRT. We also demonstrated the relationship between CAR-EBV DNA (C-E) grade and the clinical characteristics of NPC. A nomogram based on CAR-EBV DNA (C-E) grade was built for an authentic prognostic prediction in NPC patients who received CCRT in order to assist clinical work.

## Patients and Methods

### Patients and Sample Selection

In all, 842 NPC patients were recruited at the Sun Yat-sen University Cancer Center (SYSUCC; Guangzhou, China) from December 2009 to December 2014. The inclusion criteria were as follows: 1) histologic diagnosis of NPC; 2) patients who received CRRT; and 3) clinical stage II–IVa based on the 8th AJCC staging system; The exclusion criteria were: 1) synchronal malignancies; 2) lack of pretreatment peripheral blood examination and EBV DNA copy number; and 3) insufficient follow-up data. All patients involved in this study provided written informed consent.

All procedures performed in studies involving human participants were in accordance with the ethical standards of the institutional and/or national research committee and with the 1964 Helsinki declaration and its later amendments or comparable ethical standards. Informed consent was obtained from all individual participants included in the study.

All patients underwent pretreatment evaluation that included complete patient history, physical examination, hematology and biochemistry profiles, magnetic resonance imaging (MRI) of the neck and nasopharynx, chest radiography, abdominal sonography, and a whole-body bone scan using single photon-emission computed tomography. All patients’ data was from SYSUCC, and blood samples and EBV DNA copy number were obtained one week before initiation of any treatment.

### Treatment

All selected patients were treated according to the SYSUCC treatment protocol for NPC. Generally, all patients received intensity-modulated radiotherapy (IMRT) at a total dose of 68–70 Gy to the primary lesions, while metastatic lymph node areas received 2 Gy/day, 5 times per week. The regional lymphatic drainage area was irradiated with a total dose of 50–54 Gy. All patients also underwent simultaneous chemotherapy during IMRT with cisplatin, carboplatin, or nedaplatin, using either a weekly or triweekly regimen.

### Construction and Grades of CAR-EBV DNA

CAR was calculated by dividing the C-reactive protein and albumin levels. The cut-off of CAR and EBV DNA level were obtained according to the Youden index based on receiver operating characteristic (ROC) curves. Based on the cut-off values, patients with both lower CAR and EBV DNA levels were assigned grade 0, those with either increased CAR or increased EBV DNA level were assigned grade 1; and those with both higher CAR and EBV DNA level were assigned grade 2.

### Outcome and Follow-Up

Overall survival time was defined as the duration from the date of diagnosis to death or to the last follow-up. Recurrence-free survival (RFS) was defined as the time from the date of diagnosis to the date of first recurrence, death from any cause, or last follow-up. Distant metastasis-free survival (DMFS) time was defined as the period from the date of diagnosis to metastasis, death from any cause, or last follow-up. All patients were followed-up by outpatient examination or telephonic interviews.

### Statistical Analysis

All analyses were conducted using the SPSS software version 25.0 (IBM Corp., Armonk, NY); GraphPad Prism version 6.0 (GraphPad, La Jolla, CA); and R software (Version 5.1–0, Vanderbilt University, Nashville, TN). SPSS software was used to calculate the ROC curve to determine the cut-off values for CAR and EBV DNA. The relationships between CAR-EBV DNA (C-E) grade and other key clinicopathological characteristics were analyzed by chi-square test or Fisher’s exact test. Univariate and multivariate analyses were performed using the Cox proportional hazards model. Two-tailed P values <0.05 were considered to indicate statistical significance. Survival curves were plotted by the Kaplan–Meier method, and significance was determined by the log-rank test. A nomogram was generated with endpoints of 3-year and 5-year OS and DMFS, using the R software “rms” package. The concordance index (C-index) for OS and DMFS and the calibration plots were obtained. 1000 bootstrap samples and 10-fold cross-validation was also applied to avoid overfitting.

## Results

### Receiver Operating Curve of C-E Grade

**Figure 1:**
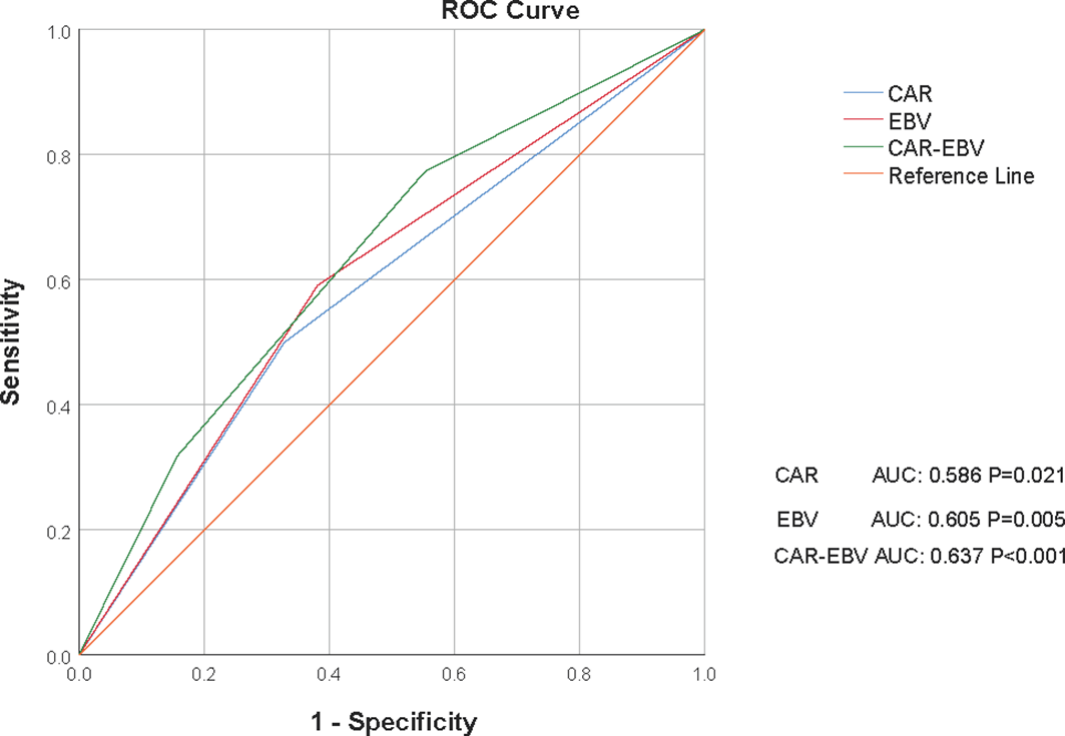
Receiver operating curves (ROCs) for CAR, EBV DNA level, and C-E grade for survival status in the whole cohort. CAR, C-reactive protein/albumin ratio; EBV, Epstein–Barr virus; C-E grade, CAR-EBV DNA grade.

According to the maximum Youden index value (**Figure 1**), the optimal cut-off value for CAR was 0.537 (AUC: 0.586, 95% CI: 0.512–0.659, P=0.021, sensitivity: 50.00%, specificity: 67.10%) and the optimal cut-off value for EBV DNA level was 2895 (AUC: 0.605, 95% CI: 0.533–0.676, P=0.005, sensitivity: 59.10%, specificity: 61.90%). The AUC for CAR-EBV DNA grade was 0.637 (95% CI: 0.568–0.706, P<0.001, sensitivity: 77.30%, specificity: 44.70%).

### Patients’ Characteristics and Relationships Between C-E Grade With Clinicopathological Features

In all, 842 NPC patients were enrolled in this study. The relationship between clinicopathological characteristics and CAR-EBV DNA of the whole cohort is presented in **Table 1**. The relationship between features with CAR and EBV DNA is given in the **Supplementary Materials**. Briefly, the median age of patients was 45 (range: 18–84) years. The male:female ratio was 2.89:1. In the pathological classification, most patients (829, 98.5%) had non-keratinizing (undifferentiated) NPC. According to the TNM staging system, 116 (13.8%) patients were in clinical stage 2, 565 (67.1%) were in clinical stage 3, and 161 (19.1%) patients were in clinical stage 4.

**Table 1:** The relationship between C-E grade and clinicopathologic characteristics in the whole cohort.

Characteristics	Total(N=842)	C-E Grade			*P*
		0	1	2
**Age (years)**					
≤45	426(50.6%)	190(22.6%)	165(19.6%)	71(8.4%)	0.629
>45	416(49.4%)	172(20.4%)	172(20.4%)	72(8.6%)
**Sex**					
Male	626(74.3%)	250(29.7%)	254(30.2%)	122(14.4%)	0.001*
Female	216(25.7%)	112(13.3%)	83(9.9%)	21(2.5%)	
**Histology**					
keratinizing	1(0.1%)	0(0%)	1(0.1%)	0(0%)	0.371
non-keratinizing (differentiated)	12 (1.4%)	7(0.8%)	2(0.2%)	3(0.4%)
non-keratinizing (undifferentiated)	829(98.5%)	355(42.2%)	334(39.7%)	140(16.6%)	
**T stage**					
1	41(4.9%)	23(2.7%)	15(1.8%)	3(0.4%)	<0.001*
2	160(19.0%)	76(9.0%)	65(7.7%)	19(2.3%)	
3	518(61.5%)	231(27.4%)	202(24.0%)	85(10.1%)	
4	123(14.6%)	32(3.8%)	55(6.5%)	36(4.3%)	
**N stage**					
0	80(9.5%)	43(5.1%)	30(3.6%)	7(0.8%)	<0.001*
1	452(53.7%)	219(26.0%)	180(21.4%)	53(6.3%)	
2	266(31.6%)	92(10.9%)	104(12.4%)	70(8.3%)	
3	44(5.2%)	8(1.0%)	23(2.7%)	13(1.5%)	
**Clinical stage**					
2	116(13.8%)	62(7.4%)	48(5.7%)	6(0.7%)	<0.001*
3	565(67.1%)	261(31.0%)	214(25.4%)	90(10.7%)	
4	161(19.1%)	39(4.6%)	75(8.9%)	47(5.6%)	

*P<0.05; C-E grade, CAR-EBV DNA grade.

The whole cohort was randomly divided into a training set and validation set (ratio: 9:1, respectively) (**Table 2**). With respect to C-E grade, 362 (43.0%), 337 (40.0%), and 143 (17.0%) patients were assigned to grade 0, grade 1, grade 2, respectively.

**Table 2:** Clinicopathologic characteristics of the patients.

Characteristics	Training set	Validation set
	(N=758)	(N=84)
**Age**		
≤45	381(45.2%)	45(5.4%)
>45	377(44.8%)	39(4.6%)
**Sex**		
Male	562(66.7%)	64(7.6%)
Female	196(23.3%)	20(2.4%)
**Histological type**		
1	1(0.1%)	0(0.0%)
2	10(1.2%)	2(0.2%)
3	747(88.7%)	82(9.8%)
**T stage^#^**		
1	36(4.3%)	5(0.6%)
2	147(17.5%)	13(1.5%)
3	469(55.7%)	49(5.8%)
4	106(12.6%)	17(2.0%)
**N stage^#^**		
0	68(8.1%)	12(1.4%)
1	407(48.4%)	45(5.3%)
2	241(28.6%)	25(3.0%)
3	42(5.0%)	2(0.2%)
**Clinical stage^#^**		
2	103(12.2%)	13(1.5%)
3	513(60.9%)	52(6.2%)
4	142(16.9%)	19(2.3%)
**CAR**		
0	495(58.8%)	59(7.0%)
1	263(31.2%)	25(3.0%)
**EBV DNA level**		
0	459(54.5%)	48(5.7%)
1	299(35.5%)	36(4.3%)
**C-E grade**		
Grade 0	322(38.2%)	40(4.8%)
Grade 1	310(36.8%)	27(3.2%)
Grade 2	126(15.0%)	17(2.0%)

CAR, C-reactive protein/albumin ratio; EBV, Epstein–Barr virus; C-E grade, CAR-EBV DNA grade.

^#^According to the 7th edition of the UICC/AJCC staging system.

### Survival Analysis of CAR, EBV DNA, and C-E Grade

**Figure 2:**
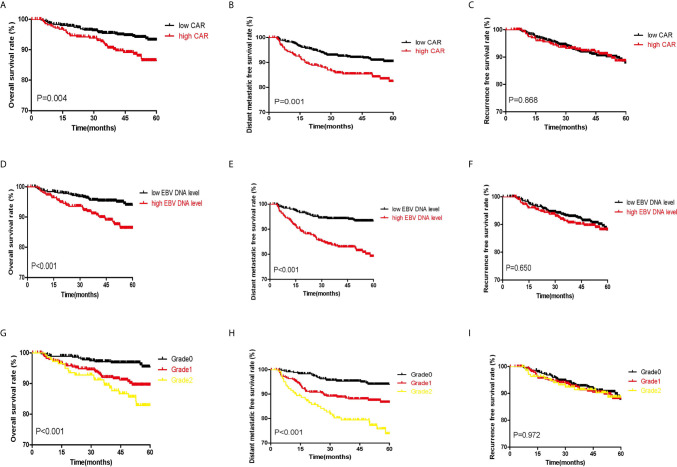
Kaplan–Meier survival curves of the whole set of patients with NPC patients. **(A–C)** show the survival curves for OS, DMFS, and RFS, respectively, according to the classification of CAR. **(D–F)** show the survival curves for OS, DMFS, and RFS, respectively, according to classification of EBV DNA copy number. **(G–I)** show the survival curves for OS, DMFS, and RFS, respectively, according to classification of C-E grade.

According to classifications of the three indicators, the whole cohort was divided into different groups. **Figure 2** shows the significant survival differences among the different groups. According to optimal cut-off and gradation, we noticed that higher CAR, EBV DNA level, and C-E grade were associated with shorter OS and DMFS (all P<0.05). The OS, DMFS, and RFS for patients in the high and low CAR groups were 46.88 and 48.78 months (P=0.004), 44.44 and 47.37 months (P=0.001), and 44.42 and 45.98 months (P=0.868), respectively (**Figures 2A–C**). The OS, DMFS, and RFS for patients in the high and low EBV DNA groups were 46.92 and 48.93 months (P<0.001), 43.97 and 47.96 months (P<0.001), and 44.75 and 45.91 months (P=0.650), respectively (**Figures 2D, E**). The OS, DMFS, and RFS for patients in the C-E grades were 46.88, 46.92, and 49.75 months (P<0.001); 43.54, 44.74, and 49.01 months (P<0.001); and 44.58, 44.61, and 46.57 months (P=0.972), respectively (**Figures 2G–I**).

Furthermore, we performed univariate and multivariate analyses for OS and DMFS. We observed that C-E grades were a significant independent prognostic factor (**Tables 3** and **4**, all P<0.05). There were no obvious differences in the survivals for RFS, we did not procedure univariate and multivariate analyses for RFS.

**Table 3:** Univariate and multivariate analyses of overall survival in the whole cohort.

Characteristics	Univariate analysis		Multivariate Cox regression analysis	
	Hazard ratio(95%CI)	*P*	Hazard ratio(95%CI)	*P*
**Age (years)**	1.552(0.950-2.535)	0.079		
**Sex**	0.777(0.431-1.402)	0.402		
**Histology**	0.426(0.157-1.156)	0.094		
**T stage**	1.611(1.111-2.337)	0.012*	1.526(1.050-2.220)	0.027*
**N stage**	1.797(1.301-2.481)	<0.001*	1.665(1.193-2.323)	0.003*
**Clinical stage**	2.095(1.366-3.213)	0.001*		
**CAR**	2.016(1.244-3.267)	0.004*		
**EBV DNA level**	2.292(1.403-3.745)	0.001*		
**C-E grade**	1.942(1.411-2.674)	<0.001*	1.621(1.193-2.323)	0.005*

*P<0.05; CAR, C-reactive protein/albumin ratio; EBV, Epstein–Barr virus; C-E grade, CAR-EBV DNA grade.

**Table 4:** Univariate and multivariate analyses of distant metastasis-free survival in the whole cohort.

Characteristics	Univariate analysis		Multivariate Cox regression analysis	
	Hazard ratio(95%CI)	*P*	Hazard ratio(95%CI)	*P*
**Age (years)**	0.986(0.653-1.487)	0.946		
**Sex**	0.822(0.501-1.350)	0.439		
**Histology**	0.533(0.191-1.483)	0.228		
**T stage**	1.164(0.863-1.570)	0.319		
**N stage**	1.843(1.398-2.429)	<0.001*	1.588(1.196-2.110)	0.001*
**Clinical stage**	1.643(1.141-2.367)	0.008*		
**CAR**	1.911(1.266-2.883)	0.002*		
**EBV DNA level**	3.134(2.032-4.836)	<0.001*		
**C-E grade**	2.144(1.632-2.816)	<0.001*	1.943(1.470-2.567)	<0.001*

*P<0.05; CAR, C-reactive protein/albumin ratio; EBV, Epstein–Barr virus; C-E grade, CAR-EBV DNA grade.

In the training set, we conducted survival analysis and univariate and multivariate analyses (see **Supplementary Materials**). The results were in line with those of the whole set. For OS and DMFS, high-level groups of CAR, EBV DNA level, and C-E grade had shorter survival time than low-level groups (all P<0.05). And no significant intergroup difference was found with respect to RFS (all P>0.05). The results of univariate and multivariate analyses were consistent with the whole set.

### Construction and Validation of the Nomogram

**Figure 3:**
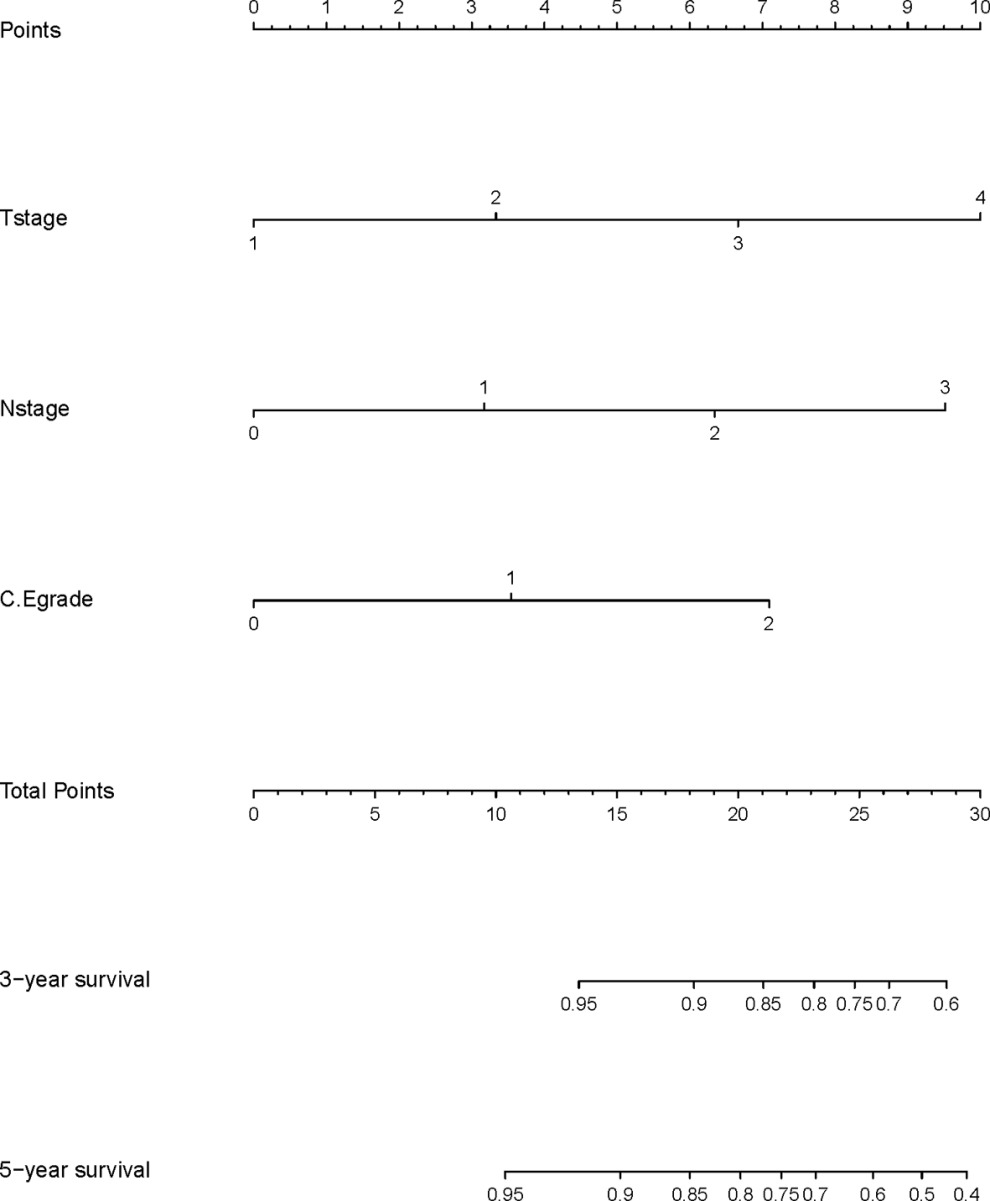
A nomogram predicting the 3- and 5-year overall survival of NPC patients.

**Figure 4:**
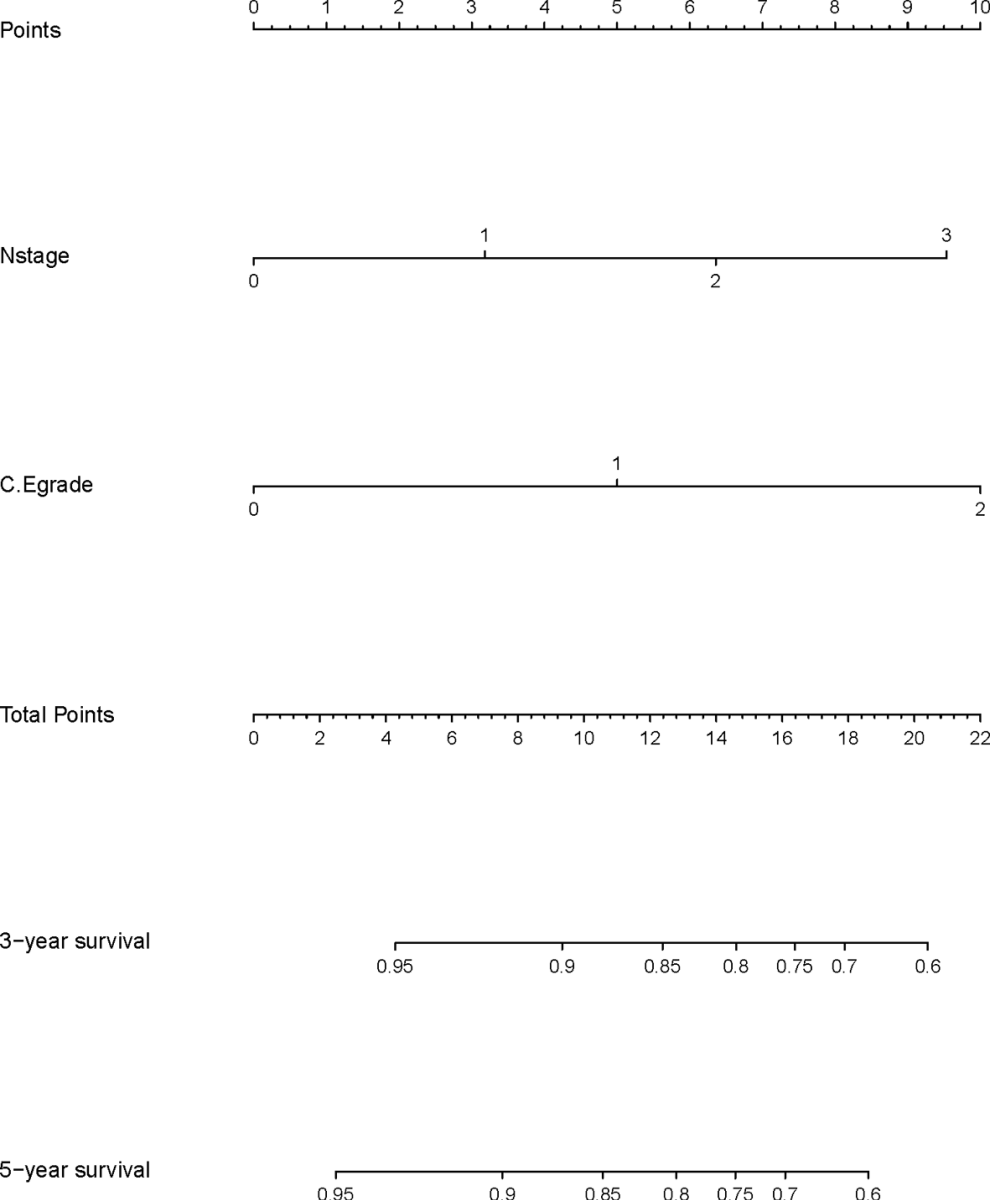
A nomogram predicting the 3- and 5-year distant metastasis-free survival of NPC patients.

A nomogram model was built based on the results of multivariate analysis. Independent prognostic indicators were integrated into the prediction of OS and DMFS times. With regard to OS time, T stage, N stage, and C-E grade were included (**Figure 3**). And for DMFS time, these factors included N stage, and C-E grade (**Figure 4**).

**Figure 5:**
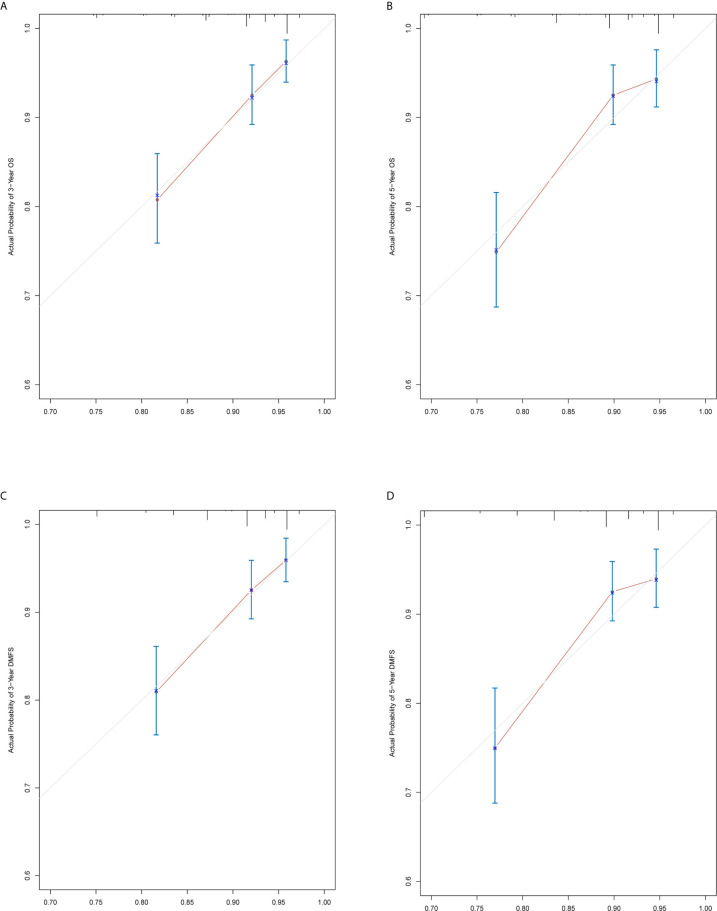
Nomogram model calibration curves of 3-year **(A)** and 5-year **(B)** overall survival, and 3-year **(C)** and 5-year **(D)** distant metastasis-free survival in the training cohort.

**Figure 6:**
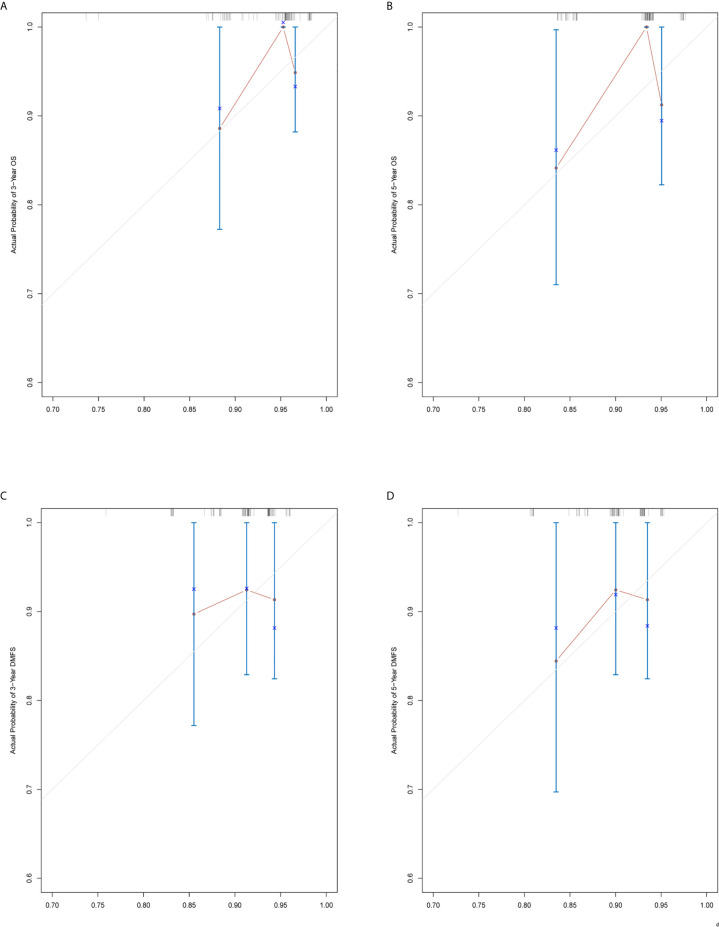
Nomogram model calibration curves of 3-year **(A)** and 5-year **(B)** overall survival, and 3-year **(C)** and 5-year **(D)** distant metastasis-free survival in the validation cohort.

The prediction ability of the final model was assessed using the C-index. For OS, the value was 0.693 (95% CI: 0.661–0.725) as well as the bias-corrected C-index, which was estimated using bootstrap with 1000 iterations and noted to be 0.695 (95% CI: 0.657, 0.733). For DMFS, the values were 0.693 (95%CI: 0.664–0.722) and 0.690 (95% CI: 0.678, 0.702), respectively. In addition, the result of internal 10-fold cross-validation for OS (C-index: 0.696, 95% CI: 0.688, 0.704) and DMFS (C-index: 0.681, 95% CI: 0.640, 0.722) also showed favorable predictive efficacy. In validation set, the C-index of the nomogram was 0.642 (95%CI: 0.527–0.757) for OS and 0.574 (95%CI: 0.462–0.686) for DMFS. The calibration curves showed good consistency with actual observation in prediction of 3- and 5-year OS and DMFS (**Figures 5** and **6**).

## Discussion

In this retrospective study, 842 NPC patients who received CCRT were included. First, we found that CAR and EBV DNA levels were independent predictors of survival in a cohort of NPC patients. By combining pretreatment CAR with EBV DNA level, we developed a novel evaluation grade and proposed a new prognostic factor—C-E grade—in NPC patients. We demonstrated that patients in different groups according to optimal cut-off had clearly distinct survival times. The univariate and multivariate analyses showed that C-E grade was an independent prognostic indicator in NPC patients. Next, we randomly divided patients into a training set and validation set. The results of the survival plot and univariate and multivariate analyses of the training set were consistent with the whole cohort. Based on the results of the multivariate analysis, a nomogram of C-E grade was constructed. Afterward, C-index and calibration in both sets were calculated, and the values confirmed the validity of this model.

In survival analysis, we noticed that the level of CAR and EBV DNA had no relationship with RFS. Actually, we are not the first research that found this trend. Several studies reported that the level of CAR was associated with OS and DMFS in NPC patients (27–30). For the level of EBV DNA, there were also some similar researches. In one research, the researchers made a conclusion that EBV DNA was more useful in detecting distant metastasis than recurrence (31). Another research investigated the relationship between EBV DNA level and survival of NPC patients, the researchers also reported that EBV DNA level was highly prognostic of long-term survival and distant metastasis in NPC patients (26). Our results were consistent with these researches that patients with higher level of CAR and EBV DNA had poorer OS and DMFS. C-E grade was obtained from CAR and EBV DNA level. Therefore, the survival curves of C-E grade were congruous with two of them so that the survival analysis of it for OS and DMFS was significant, but not for RFS. However, the underlying reason is unclear. A lot of related work need to be done to explore the differences.

Inflammatory markers have been closely associated with various tumors. For instance, Kuroda et al. showed that Controlling Nutritional Status (CONUT) is useful to not only estimate the nutritional status but also predict the long-term OS in gastric cancer patients after curative resection (32). Zhang et al. performed a meta-analysis and found that an elevated systemic immune-inflammation index (SII) predicts poor survival outcomes and is associated with clinicopathological features that indicate tumor progression of breast cancer (33). Forrest et al. found that Glasgow Prognostic Score (GPS) offers the potential to stratify patients at diagnosis and particularly for those who are being considered for active treatment in operable non-small-cell lung cancer (34). Several research studies have attested that inflammation takes part in development, recurrence, and metastasis of cancer.

C-Reactive Protein (CRP) is one of the acute-phase proteins and is synthesized by the liver (35). As an inflammatory marker, it has high sensitivity but low specificity. However, elevated CRP has been verified to relate with poor prognosis in a broad variety of cancers such as colorectal cancer, prostate cancer, and breast cancer (36). Serum albumin (ALB) is the main serum protein that can reflect the nutritional status of humans (37). Recently, many studies had found that low ALB can influence the prognosis of malignancies in different cancers such as hepatocellular carcinoma (38), colorectal cancer (39), renal cancer (40), and NPC (41). Based on the CAR value, it was clear that a high value is a risk factor for tumors. Additionally, its association with poor prognosis had been confirmed in diverse tumors (42–47) including NPC (48, 49).

Epstein–Barr virus (EBV) infection is an important etiological factor in NPC and its copy number is positively correlated with tumor stage (50). On the basis of studies, EBV DNA is widely used to screen and monitor disease change (51–53). Several studies have reported that EBV DNA level can judge the prognosis of NPC (26, 54, 55). In this study, we combined CAR and EBV DNA level as a novel biomarker to evaluate NPC prognosis. In theory, this marker could better reflect disease change and predict survival outcome. Our results showed that our hypothesis was correct, as proved by multiple statistical analyses.

We believe that C-E grade are extraordinary and comprehensive prognostic indices of NPC with respect to individual patient condition and etiology. In clinical application, C-E grade is easily available before initiation of treatment. As a risk factor, it could assist the treating clinician with an all-round and individualized understanding of the disease.

Our study has some limitations. First, selection bias was inevitable given the retrospective nature of the analysis. Second, we did not explore the relationship between C-E grade and other inflammatory indicators such as neutrophil-to-lymphocyte ratio (NLR) and platelet-to-lymphocyte ratio (PLR), which are reportedly associated with poor prognosis in NPC. Third, we concentrated on pretreatment C-E grade and did not follow-up and update the figure. It would be better to conduct a prospective study. Last, we did not validate our nomogram externally, rather only internally. Therefore, more research is required for further validation and improvement of results.

In conclusion, we proposed a new C-E grade that can be easily applied in the clinic and help clinicians to better understand the disease and formulate a treatment plan. We have shown that a high C-E grade is usually suggestive of poor prognosis. C-E grade is an independent, integrated, and personalized prognostic indicator in NPC patients. Further, the nomogram derived from C-E grade showed satisfactory predictive capacity.

## Data Availability Statement

The raw data supporting the conclusions of this article will be made available by the authors, without undue reservation.

## Ethics Statement

The studies involving human participants were reviewed and approved by Sun Yat-Sen University Cancer Center. The patients/participants provided their written informed consent to participate in this study.

## Author Contributions

Z-YY, WX, and J-JH contributed to conception and design of the study. X-WB and C-GS organized the database. XH performed the statistical analysis. Z-YH and WW wrote the first draft of the manuscript. Z-ZH, WW, and XH wrote sections of the manuscript. All authors contributed to the article and approved the submitted version.

## Conflict of Interest

The authors declare that the research was conducted in the absence of any commercial or financial relationships that could be construed as a potential conflict of interest.

The reviewers LL, LC declared a shared affiliation with the authors, to the handling editor at time of review.

## References

[1] Cao SM Simons MJ Qian CN. The Prevalence and Prevention of Nasopharyngeal Carcinoma in China. Chin J Cancer (2011) 30(2):114–9. 10.5732/cjc.010.10377 PMC4013340 21272443

[2] Parkin DM Bray F Ferlay J Pisani P. Global Cancer Statistics 2002. CA Cancer J Clin (2005) 55(2):74–108. 10.3322/canjclin.55.2.74 15761078

[3] Tsao SW Tsang CM Lo KW. Epstein-Barr Virus Infection and Nasopharyngeal Carcinoma. Philos Trans R Soc Lond B Biol Sci (2017) 372(1732). 10.1098/rstb.2016.0270 PMC5597737 28893937

[4] Lee AW Ma BB Ng WT Chan AT. Management of Nasopharyngeal Carcinoma: Current Practice and Future Perspective. J Clin Oncol (2015) 33(29):3356–64. 10.1200/JCO.2015.60.9347 26351355

[5] Zhang MX Li J Shen GP Zou X Xu JJ Jiang R. Intensity-Modulated Radiotherapy Prolongs the Survival of Patients With Nasopharyngeal Carcinoma Compared With Conventional Two-Dimensional Radiotherapy: A 10-Year Experience With a Large Cohort and Long Follow-Up. Eur J Cancer (2015a) 51(17):2587–95. 10.1016/j.ejca.2015.08.006 26318726

[6] Chen YP Chan ATC Le QT Blanchard P Sun Y Ma J. Nasopharyngeal Carcinoma. Lancet (2019) 394(10192):64–80. 10.1016/S0140-6736(19)30956-0 31178151

[7] Al-Sarraf M LeBlanc M Giri PG Fu KK Cooper J Vuong T. Chemoradiotherapy Versus Radiotherapy in Patients With Advanced Nasopharyngeal Cancer: Phase III Randomized Intergroup Study 0099. J Clin Oncol (1998) 16(4):1310–7. 10.1200/JCO.1998.16.4.1310 9552031

[8] Lin JC Jan JS Hsu CY Liang WM Jiang RS Wang WY. Phase III Study of Concurrent Chemoradiotherapy Versus Radiotherapy Alone for Advanced Nasopharyngeal Carcinoma: Positive Effect on Overall and Progression-Free Survival. J Clin Oncol (2003) 21(4):631–7. 10.1200/JCO.2003.06.158 12586799

[9] Chen QY Wen YF Guo L Liu H Huang PY Mo HY. Concurrent Chemoradiotherapy vs Radiotherapy Alone in Stage II Nasopharyngeal Carcinoma: Phase III Randomized Trial. J Natl Cancer Inst (2011) 103(23):1761–70. 10.1093/jnci/djr432 22056739

[10] Sun XS Li XY Xiao BB Liu SL Chen QY Tang LQ. Establishment and Validation of a Nomogram for Predicting the Benefit of Concurrent Chemotherapy in Stage II Nasopharyngeal Carcinoma: A Study Based on a Phase III Randomized Clinical Trial With 10-Year Follow-Up. Oral Oncol (2020a) 100:104490. 10.1016/j.oraloncology.2019.104490 31790913

[11] Sun XS Xiao BB Lin C Liu SL Chen QY Tang LQ. Establishment and Validation of Two Nomograms to Predict the Benefit of Concurrent Chemotherapy in Stage II-IVa Nasopharyngeal Carcinoma Patients With Different Risk Factors: Analysis Based on a Large Cohort. Cancer Med (2020b) 9(5):1661–70. 10.1002/cam4.2841 PMC7050092 31925942

[12] Wang HY Sun BY Zhu ZH Chang ET To KF Hwang JS. Eight-Signature Classifier for Prediction of Nasopharyngeal [Corrected] Carcinoma Survival. J Clin Oncol (2011) 29(34):4516–25. 10.1200/JCO.2010.33.7741 22025164

[13] Balkwill F Mantovani A. Inflammation and Cancer: Back to Virchow? Lancet (2001) 357(9255):539–45. 10.1016/S0140-6736(00)04046-0 11229684

[14] Coussens LM Werb Z. Inflammation and Cancer. Nature (2002) 420(6917):860–7. 10.1038/nature01322 PMC2803035 12490959

[15] Mantovani A Allavena P Sica A Balkwill F. Cancer-Related Inflammation. Nature (2008) 454(7203):436–44. 10.1038/nature07205 18650914

[16] Okada F. Inflammation and Free Radicals in Tumor Development and Progression. Redox Rep (2002) 7(6):357–68. 10.1179/135100002125001135 12625943

[17] Muller AJ Sharma MD Chandler PR Duhadaway JB Everhart ME Johnson BA 3rd. Chronic Inflammation That Facilitates Tumor Progression Creates Local Immune Suppression by Inducing Indoleamine 2,3 Dioxygenase. Proc Natl Acad Sci USA (2008) 105(44):17073–8. 10.1073/pnas.0806173105 PMC2579380 18952840

[18] Maru Y. [Inflammation in Tumor Progression]. Nihon Yakurigaku Zasshi (2011) 138(4):155–60. 10.1254/fpj.138.155 21986064

[19] Coffelt SB de Visser KE. Cancer: Inflammation Lights the Way to Metastasis. Nature (2014) 507(7490):48–9. 10.1038/nature13062 24572360

[20] Corbeau I Jacot W Guiu S. Neutrophil to Lymphocyte Ratio as Prognostic and Predictive Factor in Breast Cancer Patients: A Systematic Review. Cancers (Basel) (2020) 12(4):958. 10.3390/cancers12040958 PMC7226461 32295078

[21] Cheng CB Zhang QX Zhuang LP Sun JW. Prognostic Value of Lymphocyte-to-C-Reactive Protein Ratio in Patients With Gastric Cancer After Surgery: A Multicentre Study. Jpn J Clin Oncol (2020) 50(10):1141–9. 10.1093/jjco/hyaa099 32564084

[22] Iseki Y Shibutani M Maeda K Nagahara H Ohtani H Sugano K. Impact of the Preoperative Controlling Nutritional Status (CONUT) Score on the Survival After Curative Surgery for Colorectal Cancer. PLoS One (2015) 10(7):e0132488. 10.1371/journal.pone.0132488 26147805 PMC4492767

[23] Yan X Li G. Preoperative Systemic Immune-Inflammation Index Predicts Prognosis and Guides Clinical Treatment in Patients With non-Small Cell Lung Cancer. Biosci Rep (2020) 40(3). 10.1042/BSR20200352 PMC7103585 32175568

[24] Gundog M Basaran H. The Prognostic Value of Neutrophil-to-Lymphocyte Ratio and Platelet-to-Lymphocyte Ratio in Nasopharyngeal Cancer. J BUON (2020) 25(1):367–75. 32277656

[25] Li XH Chang H Xu BQ Tao YL Gao J Chen C. An Inflammatory Biomarker-Based Nomogram to Predict Prognosis of Patients With Nasopharyngeal Carcinoma: An Analysis of a Prospective Study. Cancer Med (2017b) 6(1):310–9. 10.1002/cam4.947 PMC5269708 27860387

[26] Zhang W Chen Y Chen L Guo R Zhou G Tang L. The Clinical Utility of Plasma Epstein-Barr Virus DNA Assays in Nasopharyngeal Carcinoma: The Dawn of a New Era?: A Systematic Review and Meta-Analysis of 7836 Cases. Med (Baltimore) (2015b) 94(20):e845. 10.1097/MD.0000000000000845 PMC4602858 25997061

[27] Zhang Y Zhou GQ Liu X Chen L Li WF Tang LL. Exploration and Validation of C-Reactive Protein/Albumin Ratio as a Novel Inflammation-Based Prognostic Marker in Nasopharyngeal Carcinoma. J Cancer (2016b) 7(11):1406–12. 10.7150/jca.15401 PMC4964124 27471556

[28] Gao N Yang RN Meng Z Wang WH. The Prognostic Value of C-Reactive Protein/Albumin Ratio in Nasopharyngeal Carcinoma: A Meta-Analysis. Biosci Rep (2018) 38(6). 10.1042/BSR20180686 PMC6239271 30352836

[29] Wang Y Yang L Xia L Chen Y. High C-Reactive Protein/Albumin Ratio Predicts Unfavorable Distant Metastasis-Free Survival in Nasopharyngeal Carcinoma: A Propensity Score-Matched Analysis. Cancer Manag Res (2018) 10:371–81. 10.2147/CMAR.S155604 PMC5827464 29503584

[30] Yang S Zhao K Ding X Jiang H Lu H. Prognostic Significance of Hematological Markers for Patients With Nasopharyngeal Carcinoma: A Meta-Analysis. J Cancer (2019) 10(11):2568–77. 10.7150/jca.26770 PMC6584332 31258763

[31] Tan R Phua SKA Soong YL Oon LLE Chan KS Lucky SS. Clinical Utility of Epstein-Barr Virus DNA and Other Liquid Biopsy Markers in Nasopharyngeal Carcinoma. Cancer Commun (Lond) (2020) 40(11):564–85. 10.1002/cac2.12100 PMC7668470 32989921

[32] Kuroda D Sawayama H Kurashige J Iwatsuki M Eto T Tokunaga R. Controlling Nutritional Status (CONUT) Score Is a Prognostic Marker for Gastric Cancer Patients After Curative Resection. Gastric Cancer (2018) 21(2):204–12. 10.1007/s10120-017-0744-3 28656485

[33] Zhang Y Sun Y Zhang Q. Prognostic Value of the Systemic Immune-Inflammation Index in Patients With Breast Cancer: A Meta-Analysis. Cancer Cell Int (2020) 20:224. 10.1186/s12935-020-01308-6 32528232 PMC7282128

[34] Forrest LM McMillan DC McArdle CS Angerson WJ Dunlop DJ. Comparison of an Inflammation-Based Prognostic Score (GPS) With Performance Status (ECOG) in Patients Receiving Platinum-Based Chemotherapy for Inoperable non-Small-Cell Lung Cancer. Br J Cancer (2004) 90(9):1704–6. 10.1038/sj.bjc.6601789 PMC2409737 15150622

[35] Pepys MB Hirschfield GM. C-Reactive Protein: A Critical Update. J Clin Invest (2003) 111(12):1805–12. 10.1172/JCI18921 PMC161431 12813013

[36] Allin KH Nordestgaard,BG. Elevated C-Reactive Protein in the Diagnosis, Prognosis, and Cause of Cancer. Crit Rev Clin Lab Sci (2011) 48(4):155–70. 10.3109/10408363.2011.599831 22035340

[37] Bauer J Capra S. Comparison of a Malnutrition Screening Tool With Subjective Global Assessment in Hospitalised Patients With Cancer–Sensitivity and Specificity. Asia Pac J Clin Nutr (2003) 12(3):257–60. 14505986

[38] Chan AW Chan SL Mo FK Wong GL Wong VW Cheung YS. Albumin-To-Alkaline Phosphatase Ratio: A Novel Prognostic Index for Hepatocellular Carcinoma. Dis Markers (2015) 2015:564057. 10.1155/2015/564057 25737613 PMC4337043

[39] Nazha B Moussaly E Zaarour M Weerasinghe C Azab B. Hypoalbuminemia in Colorectal Cancer Prognosis: Nutritional Marker or Inflammatory Surrogate? World J Gastrointest Surg (2015) 7(12):370–7. 10.4240/wjgs.v7.i12.370 PMC4691717 26730282

[40] Stenman M Laurell A Lindskog M. Prognostic Significance of Serum Albumin in Patients With Metastatic Renal Cell Carcinoma. Med Oncol (2014) 31(3):841. 10.1007/s12032-014-0841-7 24477648

[41] Yang H Wang K Liang Z Guo S Zhang P Xu Y. Prognostic Role of Pre-Treatment Serum Albumin in Patients With Nasopharyngeal Carcinoma: A Meta-Analysis and Systematic Review. Clin Otolaryngol (2020a) 45(2):167–76. 10.1111/coa.13454 31573757

[42] Xu HJ Ma Y Deng F Ju WB Sun XY Wang H. The Prognostic Value of C-Reactive Protein/Albumin Ratio in Human Malignancies: An Updated Meta-Analysis. Onco Targets Ther (2017) 10:3059–70. 10.2147/OTT.S137002 PMC5488759 28790840

[43] Chen YY Zhang JH Zhang W Yang ZK Luo RC Kang SJ. [C-Reactive Protein/Albumin Ratio as a Novel Inflammation-Based Prognostic Index for Predicting Outcomes of Patients With Colorectal Cancer]. Nan Fang Yi Ke Da Xue Xue Bao (2017) 37(5):622–7. 10.3969/j.issn.1673-4254.2017.05.09 PMC6780472 28539284

[44] Guo S He X Chen Q Yang G Yao K Dong P. The C-Reactive Protein/Albumin Ratio, a Validated Prognostic Score, Predicts Outcome of Surgical Renal Cell Carcinoma Patients. BMC Cancer (2017) 17(1):171. 10.1186/s12885-017-3119-6 28264659 PMC5339967

[45] Koh YW Lee HW. Prognostic Impact of C-Reactive Protein/Albumin Ratio on the Overall Survival of Patients With Advanced Nonsmall Cell Lung Cancers Receiving Palliative Chemotherapy. Med (Baltimore) (2017) 96(19):e6848. 10.1097/MD.0000000000006848 PMC5428608 28489774

[46] Yang X Song X Zhang L Wu C. Prognostic Role of the Pretreatment C-Reactive Protein/Albumin Ratio in Gastric Cancer: A Systematic Review and Meta-Analysis. Med (Baltimore) (2020b) 99(10):e19362. 10.1097/MD.0000000000019362 PMC7478778 32150079

[47] Liu Z Shi H Chen L. Prognostic Role of Pre-Treatment C-Reactive Protein/Albumin Ratio in Esophageal Cancer: A Meta-Analysis. BMC Cancer (2019) 19(1):1161. 10.1186/s12885-019-6373-y 31783812 PMC6884775

[48] Sun P Chen C Xia Y Bi X Liu P Zhang F. The Ratio of C-Reactive Protein/Albumin Is a Novel Inflammatory Predictor of Overall Survival in Cisplatin-Based Treated Patients With Metastatic Nasopharyngeal Carcinoma. Dis Markers (2017) 2017:6570808. 10.1155/2017/6570808 28676731 PMC5476879

[49] Yang X Liu H He M Liu M Zhou G Gong P. Prognostic Value of Pretreatment C-Reactive Protein/Albumin Ratio in Nasopharyngeal Carcinoma: A Meta-Analysis of Published Literature. Med (Baltimore) (2018) 97(30):e11574. 10.1097/MD.0000000000011574 PMC6078726 30045284

[50] Lo YM. Quantitative Analysis of Epstein-Barr Virus DNA in Plasma and Serum: Applications to Tumor Detection and Monitoring. Ann N Y Acad Sci (2001) 945:68–72. 10.1111/j.1749-6632.2001.tb03865.x 11708496

[51] Leung SF Zee B Ma BB Hui EP Mo F Lai M. Plasma Epstein-Barr Viral Deoxyribonucleic Acid Quantitation Complements Tumor-Node-Metastasis Staging Prognostication in Nasopharyngeal Carcinoma. J Clin Oncol (2006) 24(34):5414–8. 10.1200/JCO.2006.07.7982 17135642

[52] Chan KC. Plasma Epstein-Barr Virus DNA as a Biomarker for Nasopharyngeal Carcinoma. Chin J Cancer (2014) 33(12):598–603. 10.5732/cjc.014.10192 25418194 PMC4308655

[53] Yip TT Ngan RK Fong AH Law SC. Application of Circulating Plasma/Serum EBV DNA in the Clinical Management of Nasopharyngeal Carcinoma. Oral Oncol (2014) 50(6):527–38. 10.1016/j.oraloncology.2013.12.011 24440146

[54] Zhang J Shu C Song Y Li Q Huang J Ma X. Epstein-Barr Virus DNA Level as a Novel Prognostic Factor in Nasopharyngeal Carcinoma: A Meta-Analysis. Med (Baltimore) (2016a) 95(40):e5130. 10.1097/MD.0000000000005130 PMC5059099 27749596

[55] Li WF Zhang Y Huang XB Du XJ Tang LL Chen L. Prognostic Value of Plasma Epstein-Barr Virus DNA Level During Posttreatment Follow-Up in the Patients With Nasopharyngeal Carcinoma Having Undergone Intensity-Modulated Radiotherapy. Chin J Cancer (2017a) 36(1):87. 10.1186/s40880-017-0256-x 29116021 PMC5678814

