# Supplementary material for: Establishment and Validation of Nomogram Based on Combination of Pretreatment C-Reactive Protein/Albumin Ratio–EBV DNA Grade in Nasopharyngeal Carcinoma Patients Who Received Concurrent Chemoradiotherapy
Source: Front Oncol. 2021 Jul 15;11:583283. doi: 10.3389/fonc.2021.583283 (PMC8320887; doi:10.3389/fonc.2021.583283)
Supplement: Supplementary file 1 [file DataSheet_1.docx]

**Supplementary Materials**

**Supplementary Table 1**. The relationship between CAR and clinicopathologic characteristics in the whole cohort.

| **Characteristics** | | **Total**  **(N=842)** | **CAR** | | ***P*** |
| --- | --- | --- | --- | --- | --- |
|  |  |  | **Score 0** | **Score 1** |  |
| **Age (years)** |  |  |  |  |  |
| ≤45 | | 426(50.6%) | 292(34.7%) | 134(15.9%) | 0.089 |
| ＞45 | | 416(49.4%) | 262(31.1%) | 154(18.3%) |  |
| **Sex** | |  |  |  |  |
| Male | | 626(74.3%) | 394(46.8%) | 232(27.5%) | 0.003* |
| Female | | 216(25.7%) | 160(19.0%) | 56(6.7%) |  |
| **Histology** | |  |  |  |  |
| 1 | | 1(0.1%) | 1(0.1%) | 0(0%) | 0.769 |
| 2 | | 12 (1.4%) | 8(1.0%) | 4(0.4%) |  |
| 3 | | 829(98.5%) | 545(64.8%) | 284(33.7%) |  |
| **T stage** |  |  |  |  |  |
| 1 | | 41(4.9%) | 32(3.8%) | 9(1.1%) | <0.001* |
| 2 | | 160(19.0%) | 114(13.5%) | 46(5.5%) |  |
| 3 | | 518(61.5%) | 348(41.3%) | 170(20.2%) |  |
| 4 | | 123(14.6%) | 60(7.1%) | 63(7.5%) |  |
| **N stage** |  |  |  |  |  |
| 0 | | 80(9.5%) | 52(6.2%) | 28(3.3%) | 0.003* |
| 1 | | 452(53.7%) | 321(38.1%) | 131(15.6%) |  |
| 2 | | 266(31.6%) | 158(18.8%) | 108(12.8%) |  |
| 3 | | 44(5.2%) | 23(2.7%) | 21(2.5%) |  |
| **Clinical stage** | |  |  |  |  |
| 2 | | 116(13.8%) | 87(10.4%) | 29(3.4%) | <0.001* |
| 3 | | 565(67.1%) | 387(46.0%) | 178(21.1%) |  |
| 4 | | 161(19.1%) | 80(9.5%) | 81(9.6%) |  |

**P*<0.05; Abbreviations: CAR: C-reactive protein/albumin ratio.

**Supplementary Table 2**. The relationship between EBV and clinicopathologic characteristics in the whole cohort.

| **Characteristics** | | **Total**  **(N=842)** | **EBV DNA level** | | ***P*** |
| --- | --- | --- | --- | --- | --- |
|  |  |  | **Score 0** | **Score 1** |  |
| **Age (years)** |  |  |  |  |  |
| ≤45 | | 426(50.6%) | 253(23.1%) | 173(20.5%) | 0.621 |
| ＞45 | | 416(49.4%) | 254(30.2%) | 162(19.2%) |  |
| **Sex** | |  |  |  |  |
| Male | | 626(74.3%) | 360(42.8%) | 266(31.5%) | 0.006* |
| Female | | 216(25.7%) | 147(17.5%) | 69(8.2%) |  |
| **Histology** | |  |  |  |  |
| 1 | | 1(0.1%) | 0(0%) | 1(0.1%) | 0.423 |
| 2 | | 12 (1.4%) | 8(1.0%) | 4(0.4%) |  |
| 3 | | 829(98.5%) | 499(59.3%) | 330(39.2%) |  |
| **T stage** |  |  |  |  |  |
| 1 | | 41(4.9%) | 29(3.5%) | 12(1.4%) | 0.012* |
| 2 | | 160(19.0%) | 103(12.2%) | 57(6.8%) |  |
| 3 | | 518(61.5%) | 316(37.5%) | 202(24.0%) |  |
| 4 | | 123(14.6%) | 59(7.0%) | 64(7.6%) |  |
| **N stage** |  |  |  |  |  |
| 0 | | 80(9.5%) | 64(7.6%) | 16(1.9%) | <0.001* |
| 1 | | 452(53.7%) | 297(35.3%) | 155(18.4%) |  |
| 2 | | 266(31.6%) | 130(15.4%) | 136(16.2%) |  |
| 3 | | 44(5.2%) | 16(1.9%) | 28(3.3%) |  |
| **Clinical stage** | |  |  |  |  |
| 2 | | 116(13.8%) | 85(10.1%) | 31(3.7%) | <0.001* |
| 3 | | 565(67.1%) | 349(41.4%) | 216(25.7%) |  |
| 4 | | 161(19.1%) | 73(8.7%) | 88(10.4%) |  |

**P*<0.05. Abbreviations: Epstein–Barr virus (EBV).

**Supplementary Table 3.** Univariate and multivariate analyses of overall survival in the training cohort.

| **Characteristics** | **Univariate analysis** |  | **Multivariate Cox regression analysis** |  |
| --- | --- | --- | --- | --- |
|  | Hazard ratio(95%CI) | *P* | Hazard ratio(95%CI) | *P* |
| **Age (years)** | 1.708(1.002-2.911) | 0.049* | 1.680(0.985-2.865) | 0.057 |
| **Sex** | 0.868(0.468-1.612) | 0.655 |  |  |
| **Histology** | 0.376(0.139-1.022) | 0.055 |  |  |
| **T stage** | 1.793(1.187-2.708) | 0.006* | 1.635(1.075-2.487) | 0.022* |
| **N stage** | 1.758(1.242-2.489) | 0.001* | 1.612(1.128-2.303) | 0.009* |
| **Clinical stage** | 2.206(1.383-3.517) | 0.001* |  |  |
| **CAR** | 2.149(1.277-3.614) | 0.004* |  |  |
| **EBV DNA level** | 2.527(1.483-4.308) | 0.001* |  |  |
| **C-E grade** | 2.083(1.474-2.945) | <0.001* | 1.716(1.192-2.472) | 0.004* |

**P*<0.05; Abbreviations: CAR: C-reactive protein/albumin ratio, Epstein–Barr virus (EBV), C-E grade: CAR-EBV DNA grade.

**Supplementary Table 4.** Univariate and multivariate analyses of distant metastasis-free survival in training cohort.

| **Characteristics** | **Univariate analysis** |  | **Multivariate Cox regression analysis** |  |
| --- | --- | --- | --- | --- |
|  | Hazard ratio(95%CI) | *P* | Hazard ratio(95%CI) | *P* |
| **Age (years)** | 1.022(0.664-1.571) | 0.923 |  |  |
| **Sex** | 0.806(0.478-1.358) | 0.418 |  |  |
| **Histology** | 0.472(0.174-1.280) | 0.140 |  |  |
| **T stage** | 1.113(0.813-1.523) | 0.504 |  |  |
| **N stage** | 1.855(1.392-2.473) | <0.001* | 1.573(1.170-2.114) | 0.003* |
| **Clinical stage** | 1.580(1.075-2.323) | 0.020* |  |  |
| **CAR** | 2.039(1.326-3.135) | 0.001* |  |  |
| **EBV DNA level** | 3.214(2.039-5.067) | <0.001* |  |  |
| **C-E grade** | 2.254(1.689-3.009) | <0.001* | 2.039(1.517-2.114) | <0.001* |

**P*<0.05; Abbreviations: CAR: C-reactive protein/albumin ratio, Epstein–Barr virus (EBV), C-E grade: CAR-EBV DNA grade.

Figure 1


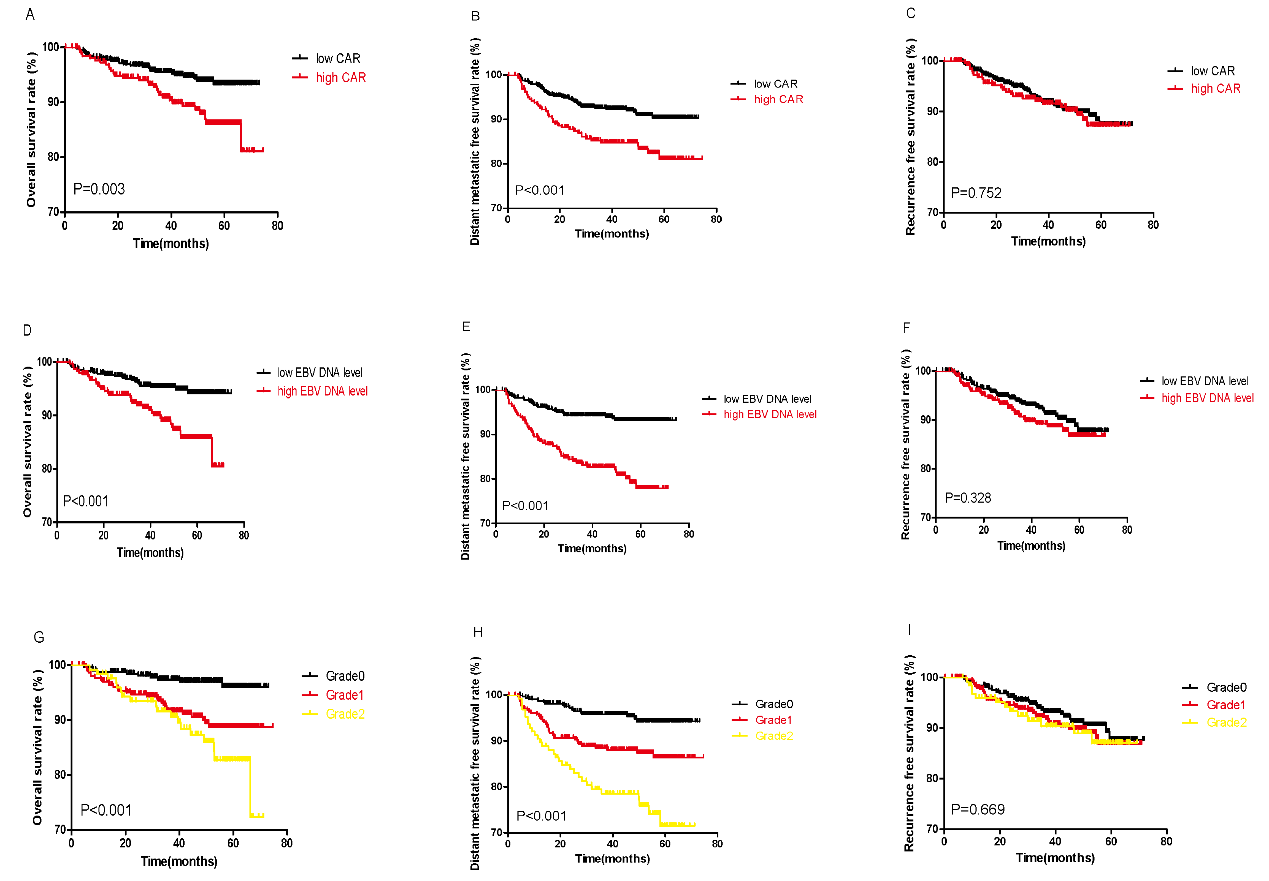


Kaplan–Meier survival curves of the training set of patients with NPC. Figures A–C show the survival curves for OS, DMFS, and RFS, respectively, according to classification of CAR. Figures D–F show the survival curves for OS, DMFS, and RFS, respectively, according to classification of EBV DNA level. Figures G–I show the survival curves for OS, DMFS, and RFS. respectively, according to classification of C-E grade.
